# [Retracted] Tissue‑specific transplantation antigen P35B functions as an oncogene and is regulated by microRNA‑125a‑5p in lung cancer

**DOI:** 10.3892/or.2024.8735

**Published:** 2024-04-15

**Authors:** Yingjie Gao, Guangliang Zhang, Jinlong Liu, Huimin Li

Oncol Rep 45: 72, 2021; DOI: 10.3892/or.2021.8023

Following the publication of this paper, it was drawn to the Editor's attention by a concerned reader that certain of the immunohistochemical data shown in Fig. 1A on p. 5, colony formation data shown in Figs. 2C, H and M and 6D on p. 6 and p. 10 respectively, the western blots in Fig. 2B, Transwell cell migration and invasion assay data in Fig. 3B, D and F, and immunofluorescence data in Fig. 4C had already appeared in previously published articles written by different authors at different research institutes (some of which have subsequently been retracted). Owing to the fact that the contentious data in the above article had already been published prior to its submission to *Oncology Reports*, the Editor has decided that this paper should be retracted from the Journal. After having been in contact with the authors, they accepted the decision to retract the paper. The Editor apologizes to the readership for any inconvenience caused.

